# 2,2′-Azobis (2-Amidinopropane) Dihydrochloride Is a Useful Tool to Impair Lung Function in Rats

**DOI:** 10.3389/fphys.2016.00475

**Published:** 2016-10-20

**Authors:** Maria D. Moreira Gomes, Giovanna M. C. Carvalho, Natalia V. Casquilho, Andressa C. P. Araújo, Samuel S. Valença, Jose H. Leal-Cardoso, Walter A. Zin

**Affiliations:** ^1^Laboratory of Respiration Physiology, Carlos Chagas Filho Institute of Biophysics, Universidade Federal do Rio de JaneiroRio de Janeiro, Brazil; ^2^Electrophysiology Laboratory, Superior Institute of Biomedical Sciences, State University of CearáFortaleza, Brazil; ^3^Biomedical Sciences Institute, Universidade Federal do Rio de JaneiroRio de Janeiro, Brazil

**Keywords:** inflammation, lung mechanics, oxidative stress, AAPH, oxidative damage

## Abstract

Recently, several studies have reported that respiratory disease may be associated with an increased production of free radicals. In this context, 2,2′-azobis (2-amidinopropane) dihydrochloride (AAPH) is a free radical-generating compound widely used to mimic the oxidative stress state. We aimed to investigate whether AAPH can generate lung functional, inflammatory, histological and biochemical impairments in the lung. Wistar rats were divided into five groups and instilled with saline solution (714 μL/kg, CTRL group) or different amounts of AAPH (25, 50, 100, and 200 mg/kg, 714 μL/kg, AAPH groups). Seventy-two hours later the animals were anesthetized, paralyzed, intubated and static elastance (Est), viscoelastic component of elastance (ΔE), resistive (ΔP1) and viscoelastic (ΔP2) pressures were measured. Oxidative damage, inflammatory markers and lung morphometry were analyzed. ΔP1 and Est were significantly higher in AAPH100 and AAPH200 than in the other groups. The bronchoconstriction indexes were larger in AAPH groups than in CTRL. The area occupied by collagen and elastic fibers, polymorpho- and mononuclear cells, malondialdehyde and carbonyl groups levels were significantly higher in AAPH200 than in CTRL. In comparison to CTRL, AAPH200 showed significant decrease and increase in the activities of superoxide dismutase and catalase, respectively. AAPH augmented the release of pro-inflammatory cytokines IL-1β, IL-6 e TNF-α. Hence, exposure to AAPH caused significant inflammatory alterations and redox imbalance accompanied by altered lung mechanics and histology. Furthermore, we disclosed that exposure to AAPH may represent a useful *in vivo* tool to trigger lung lesions.

## Introduction

Recently, several studies have reported that increased free radicals production may be associated with respiratory diseases, such as asthma, pulmonar fibrosis, chronic obstructive pulmonary disease (COPD), acute lung injury (ALI) and lung cancer (Valko et al., 2007; Ciencewicki et al., 2008; Park et al., 2009). In this line, the lung is continuously exposed to high levels of oxidants, which together with its large surface area and blood supply render it prone to injury mediated by free radicals (Rahman, 2002, 2012; Park et al., 2009).

The mechanical properties of lung tissue are important determinants of the overall mechanical behavior of the respiratory system, contributing significantly to elastic, resistive, and viscoelastic properties of the lung (Faffe and Zin, 2009; Suki et al., 2011). During disease states, mechanical properties can be altered considerably, which contributes importantly to a decline in lung function (Suki et al., 2003; Bates et al., 2007; Gladysheva et al., 2010). Several mechanisms have been suggested to explain the functional decline in lungs, such as thickened alveolar membranes (Rocco et al., 2001), increased cellularity (Casquilho et al., 2011; Oliveira et al., 2015), extracellular matrix remodeling (Rocco et al., 2001) and fibrosis (Sansores et al., 1966). In the context, prior studies report changes in lung resistance and elastance resulting from lung diseases in which oxidative stress plays a key role (Suki et al., 2003; Bezerra et al., 2011; Lima-Trajano et al., 2011).

Numerous compounds induce oxidative stress, as expressed by redox decomposition of hydroperoxides or hydrogen peroxide by metal ions, and thermal decomposition of free radical initiators, including hyponitrites, peroxides and azo compounds (Terao and Niki, 1986; Dooley et al., 1990; Yokozawa et al., 2000). In this study, 2,2′-azobis (2-amidinopropane) dihydrochloride (AAPH) was chosen as the free radical generating system to impair lung function. This water-soluble azo compound directly generates peroxyl radicals (Dooley et al., 1990; Krainev and Bigelow, 1996; Musialik et al., 2008) at physiological temperature and at a constant and reproducible rate (Fiorentini et al., 1999) without generating H_2_O_2_ (hydrogen pexoxide) as an intermediate, thus rendering its effect independent of tissue content of enzymatic antioxidants. The half-life of AAPH is about 175 h (37°C at neutral pH), allowing the rate of free radical generation to be essentially constant during the first several hours in solution (Landi et al., 1995; Peluso et al., 2002). Because of all these advantages, the AAPH has been used extensively over the years as a predictable and controllable source of free radicals, to mimic the oxidative stress and assess the free-radical-scavenging activity of drugs (Terao and Niki, 1986). Indeed, in *in vitro* experimental methods, AAPH induces hemolysis of human erythrocytes (Qasim and Mahmood, 2015), lipid peroxidation of rat thymocytes and liver (Landi et al., 1995; Cai et al., 2003) and inhibition of spontaneous motility and contractile responses of guinea pig ileum and rabbit jejunum (Peluso et al., 2002). Furthermore, AAPH has been used in *in vivo* model of oxidative stress. In this line, induction of colitis in rats by intrarectal administration of AAPH, causes erythema, edema and histologically mucosal inflammation (Tamai et al., 1992). Intraperitoneal administration of AAPH can induce lipid peroxidation in liver, kidney and heart of rats (Dooley et al., 1990). Recently, a rat model of acute pancreatitis induced by AAPH has been proven useful to investigate the early events of oxidative stress insult to the pancreas (Tukaj et al., 2012).

Although AAPH was used *in vivo*, to our knowledge there is no study in the literature concerning the pulmonary outcomes of the intranasal administration of AAPH. Hence, we aimed at evaluating whether the exposure to AAPH impairs lung functional, morphological and biochemical profiles.

## Materials and methods

### Animal preparation

Forty male Wistar rats (190–210 g) were housed in plastic cages with absorbent bedding material, maintained on a 12-h daylight cycle and randomly divided into 5 groups. Control (CTRL; *n* = 8) and experimental (AAPH; *n* = 8/group) animals received one intranasal instillation (*i.n*.) of sterile saline solution (0.9% NaCl, 37°C) or different amounts of AAPH (25, 50, 100 and 200 mg/kg in saline solution, respectively). These doses were chosen based on preliminary experiments (data not shown), which also allowed the determination of the volume of saline solution (714 μL/kg body wt) in which the highest dose of AAPH would be completely diluted. Thus, all groups received a total volume of 714 μL/kg body wt. Just before each intranasal administration, AAPH (Sigma Chemical, St. Louis, MO, USA) was diluted in saline and sonicated for 50 s. During instillation, all animals were anesthetized with sevoflurane. This procedure resulted in no deaths.

All animals received humane care in compliance with the “Principles of Laboratory Animal Care” formulated by the National Society for Medical Research, the “Guide for the Care and Use of Laboratory Animals” prepared by the National Academy of Sciences, USA, the “APS's Guiding Principles in the Care and Use of Vertebrate Animals in Research and Training,” and the National Council for Controlling Animal Experimentation, Ministry of Science, Technology and Innovation (CONCEA/MCTI), Brazil. The experiments were approved by the Ethics Committee on the Use of Animals, Health Sciences Center, Federal University of Rio de Janeiro (Protocol IBCCF 128).

### Pulmonary mechanics

Seventy-two hours after instillation, the animals were sedated with diazepam (5 mg *i.p*.), anesthetized with pentobarbital sodium (20 mg·kg body weight^−1^ *i.p*.), placed in the supine position on a surgical table, tracheotomized, and a snugly fitting cannula (1.7 mm ID) was introduced into the trachea. The animals were then paralyzed with pancuronium bromide (0.1 mg·kg body weight^−1^ *i.v*.), and mechanically ventilated (Samay VR15, Universidad de la Republica, Montevideo, Uruguay) with a frequency of 80 breaths·min^−1^, tidal volume of 1.5 mL, flow of 8 mL·s^−1^, and positive end-expiratory pressure (PEEP) of 2 cm H_2_O. The anterior chest wall was surgically removed.

A pneumotachograph (1.5 mm ID, length = 4.2 cm, distance between side ports = 2.1 cm) was connected to the tracheal cannula for the measurements of airflow (V′), and changes in lung volume (V_T_) were obtained by digital integration of the flow signal. A Validyne MP45-2 differential pressure transducer (Engineering Corp, Northridge, CA, USA) measured the pressure gradient across the pneumotachograph. The flow resistance of the equipment (Req), tracheal cannula included, constant up to flow rates of 26 ml s^−1^, amounted to 0.12 cm H_2_O.ml^−1^.s Equipment resistive pressure (= R_eq_·V′) was subtracted from pulmonary resistive pressures so that the present results represent intrinsic values. Transpulmonary pressure (P_L_) was determined by a Validyne MP-45 differential pressure transducer (Engineering Corp, Northridge, CA, USA). Lung resistive (ΔP1) and viscoelastic/inhomogeneous (ΔP2) pressures, lung static (Est) and dynamic (Edyn) elastances, as well as elastic component of viscoelasticity (ΔE) were computed by the end-inflation occlusion method (Bates et al., 1985, 1988). Briefly, after end-inspiratory occlusion, there is an initial fast drop in PL (ΔP1) from the pre-occlusion value down to an inflection point (Pi) followed by a slow pressure decay (ΔP2), until an apparent plateau is reached. This plateau corresponds to the elastic recoil pressure of the lung (Pel). ΔP1 represents the pressure used to overcome Newtonian pulmonary resistances in normal animals and humans, and ΔP2 reflects stress relaxation, or viscoelastic properties of the lung, together with a small contribution of time-constant inhomogeneities (pendelluft) (Bates et al., 1988; Saldiva et al., 1992). Lung static (Est) and dynamic elastances (Edyn) were calculated by dividing Pel and Pi by V_T_, respectively. ΔE was calculated as Est—Edyn (Bates et al., 1985, 1988). Lung mechanics was measured 10–15 times in each animal. All data were analyzed using ANADAT data analysis software (RHT-InfoData Inc., Montreal, QC, Canada).

### Histological study

Heparin (1000 IU) was intravenously injected immediately after the determination of respiratory mechanics. The trachea was clamped at end-expiration, and the abdominal aorta and vena cava were sectioned, yielding a massive hemorrhage that quickly euthanized the animals. The lungs were perfused with saline and, then, removed *en bloc*. The right lung was isolated, frozen in liquid nitrogen, and stored for further analysis; the left lung was kept at functional residual capacity and fixed in Millonig's formaldehyde (100 mL HCHO, 900 mL H_2_O, 18.6 g NaH_2_PO_4_, 4.2 g NaOH). After 24 h of fixation, specimens were dehydrated in alcohol and embedded in paraffin. Four-mm-thick slices were then cut and stained with hematoxylin-eosin (H & E) and specifically stained for collagen and elastic fibers. An investigator, who was unaware of the origin of the coded material, examined the samples microscopically.

The bronchoconstriction index (BCI) was determined with an integrating eyepiece with a coherent system with 100 points and 50 lines coupled to a conventional light microscope (Axioplan, Zeiss, Oberkochen, Germany). The point-counting technique was used to evaluate BCI, by dividing the number of intercepts with the epithelial basal membrane (NI) by the square root of the number of points (NP) falling on the airway lumen applying the equation: BCI = NI/√NP (Sakae et al., 1994). Only airways in which the long diameter did not exceed the short diameter by more than 20% were accepted for measurement. BCI evaluation was performed at 400x magnification across 5–15 random non-coincident microscopic fields in each animal.

Lung parenchyma also underwent specific staining methods to quantify collagen (Picrosirius method; Montes, 1996) and elastic fibers (Weigert's resorcin fuchsin method with oxidation; Fullmer et al., 1974) on images captured in a blinded manner across ten random non-coincident fields (400x magnification). The total tissue area of each field was also computed. The quantification was determined on captured high quality images (2048 × 1536 pixels) using the Image Pro Plus 4.5.1 software (Media Cybernetics, Silver Spring, MD, USA). A single observer blindly performed the morphological measurements. Results are expressed as fiber area/tissue area.

Slides stained with H & E were digitized for subsequent quantification of cellularity. Polymorpho- (PMN) and mononuclear (MN) cells expressed as cells/pulmonar tissue area (1000x) were counted and expressed as percentage of the cells in a defined square area (total area of 10,000 μm^2^/field) (Gundersen et al., 1988). The analysis was performed using the software Panoramic Viewer 1.15.14 (3DHISTECH Ltd, Budapest, Hungary).

### Lung homogenates

The right lungs of each group were homogenized in 1.0 mL potassium phosphate buffer (pH 7.5) and then were centrifuged at 600 g for 10 min at −4°C. The supernatants were stored at −80°C for the analysis of oxidative damage (lipid peroxidation and carbonyl assays) and antioxidant enzymes activities (catalase and superoxide dismutase). The total protein in the samples was determined by the Bradford's method (Bradford, 1976). A spectrophotometer was used in all determinations (Ultrospec 2100 pro, Amersham-Biosciences, Buckinghamshire, UK).

### Catalase and superoxide dismutase activities

Catalase (CAT) activity was measured at 240 nm by the rate of decrease in hydrogen peroxide concentration and was expressed as CAT equivalents (U/mg protein) (Aebi, 1984). Superoxide dismutase (SOD) activity was assayed by measuring inhibition of adrenaline auto-oxidation as absorbance at 480 nm and was expressed as SOD equivalents (U/mg protein) (Bannister and Calabrese, 1987).

### Oxidative damage

Oxidative damage was evaluated by quantifying malondialdehyde and protein carbonyls. As an index of oxidative damage induced by lipid peroxidation, we used the thiobarbituric acid reactive substances (TBARS) method to measure malondialdehyde (MDA) products during an acid heating reaction (Draper and Hadley, 1990). Briefly, samples were mixed with 1 ml of 10% trichloroacetic acid and 1 ml of 0.67% thiobarbituric acid. The samples were then heated in a boiling water bath for 30 min. MDA levels were determined by absorbance at 532 nm and expressed as MDA equivalents (MDA nmol/mg protein). Protein carbonyls were determined using the 2,4-dinitrophenylhydrazine (DNPH) spectrophotometry method, as previously described (Levine et al., 1990). In brief, samples containing either 2 N hydrochloric acid or DNPH were passed through columns containing Sephadex G-10 (MP Biomedicals, Santa Ana, CA, USA) and rinsed with 2 N hydrochloric acid. The difference in absorbance with and without DNPH was calculated in all samples. Carbonyl levels were determined by absorbance at 370 nm and expressed as carbonyl group equivalents (carbonyl nmol/mg protein).

### Assay of pro-inflammatory mediators

Another 25 rats (190–210 g, *n* = 6/group) underwent the same protocol and group assignment as aforementioned. The right lungs of each group were homogenized in 1.0 mL potassium phosphate buffer (pH 7.5) and then were centrifuged at 7000 g for 15 min at −4°C. The levels of pro-inflammatory mediators (TNF-α, IL-1β, and IL-6) were measured in lung homogenates by ELISA with high sensitivity kits (R&D Systems Inc., Minneapolis, MN, USA) in accordance with the manufacturer's instructions. The detection limits of this method correspond to 5.1, 3.0, or 1.6 pg mL^−1^, respectively.

### Statistical analysis

SigmaPlot 11 statistical package (SYSTAT Software, Chicago, IL, USA) was used. When percentage values were to be tested, they first underwent an arcsine transformation. The normality of the data (Kolmogorov-Smirnov test with Lilliefors' correction) and the homogeneity of variances (Levene median test) were tested. If both conditions were satisfied, one-way ANOVA test followed by Holm-Sidak test was used to assess differences among groups. If one or both conditions was/were not satisfied, Kruskal-Wallis ANOVA was used followed by Dunn's test. The significance level was set at 5%.

## Results

**Figure 1:**
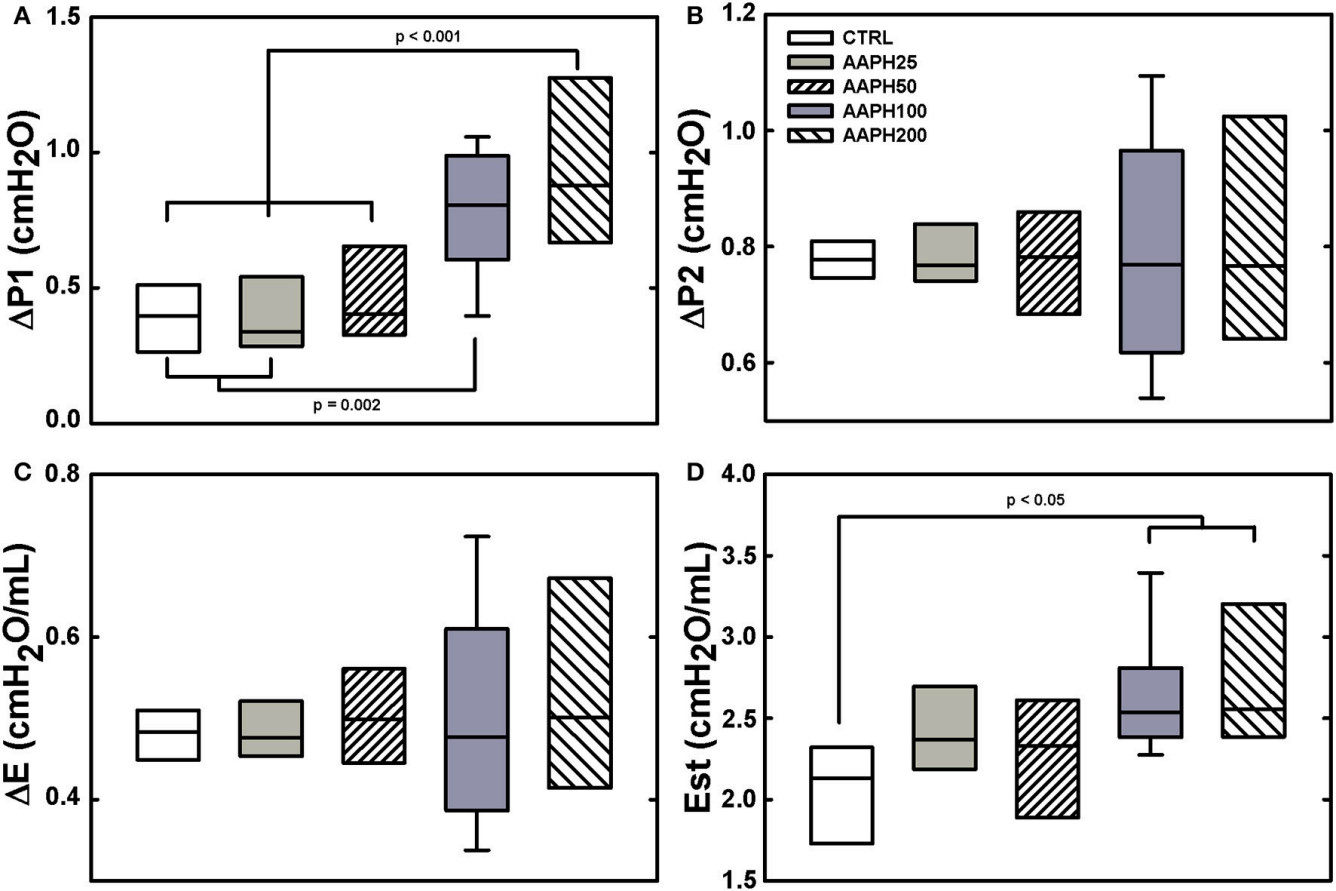
**Pulmonary mechanical data in rats. (A)** Resistive pressure (ΔP1). **(B)** Pressure dissipated to overcome viscoelastic/inhomogeneous mechanical components (ΔP2). **(C)** Elastic component of viscoelasticity (ΔE). **(D)** Static elastance (Est). Animals exposed to saline solution (CTRL) or different amounts of 2,2′-azobis (2-amidinopropane) dihydrochloride (AAPH25, 50, 100, or 200 mg/kg in saline solution). Boxes show interquartile (25–75%) range, whiskers encompass 10–90% range, and horizontal lines represent median of 8 animals in each group. Differences among the groups are indicated by horizontal lines, above which the respective *P*-values can be found.

ΔP1 was significantly higher in AAPH100 and AAPH200 rats than in CTRL and AAPH25 groups that did not differ among them. Additionally, AAPH200 was also higher than AAPH50 (Figure 1A). Accordingly, a higher resistive pressure (that reflects Newtonian resistence) suggest an increase in airway resistance to airflow. Est was larger in AAPH100 and AAPH200 animals than in the CTRL group (Figure 1D), indicating stiffer lungs. Intranasal instillation of AAPH did not alter ΔP2 and ΔE (Figures 1B,C, respectively), which means that lung tissue viscoelastic properties were not substantially altered. Bronchoconstriction index showed a significant increase in all AAPH groups compared to CTRL (Table 1), which supports the higher pressure used to overcome central airway resistance (ΔP1 seen in Figure 1).

**Table 1:** **Lung histology in rats exposed to 2,2′-azobis (2-amidinopropane) dihydrochloride (AAPH)**.

**Groups**	**BCI**	**Elastic fibers (%)**	**Collagen fibers (%)**	**PMN (cells^−2^/μm^2^)**	**MN (cells^−2^/μm^2^)**
CTRL	3.232 ± 0.194	8.155 ± 1.573	2.863 ± 0.824	1.350 ± 0.216	0.163 ± 0.032
AAPH25	4.150 ± 0.385^*^	9.017 ± 2.429	2.911 ± 0.470	1.480 ± 0.202	0.245 ± 0.052
AAPH50	4.199 ± 0.461^*^	11.017 ± 2.110	3.686 ± 0.931	1.570 ± 0.221	0.234 ± 0.114
AAPH100	4.280 ± 0.462^*^	11.439 ± 1.336	10.125 ± 2.921^*^	1.600 ± 0.196	0.245 ± 0.107
AAPH200	4.148 ± 0.556^*^	12.239 ± 2.663^*^	10.894 ± 1.878^*^	2.400 ± 0.463^*^	0.510 ± 0.151^*^

*Values are mean ± SD at 72 h after the exposure of animals to saline solution (CTRL) or different amounts of AAPH (25, 50, 100, and 200 mg/kg in saline solution). BCI: bronchoconstriction index. Elastic and collagen fibers are expressed as % surface density of fibers per tissue area in each field. PMN and MN, polymorpho- and mononuclear cells, respectively*.

* *Statistically significant different (p < 0.05) from other groups*.

**Figure 2:**
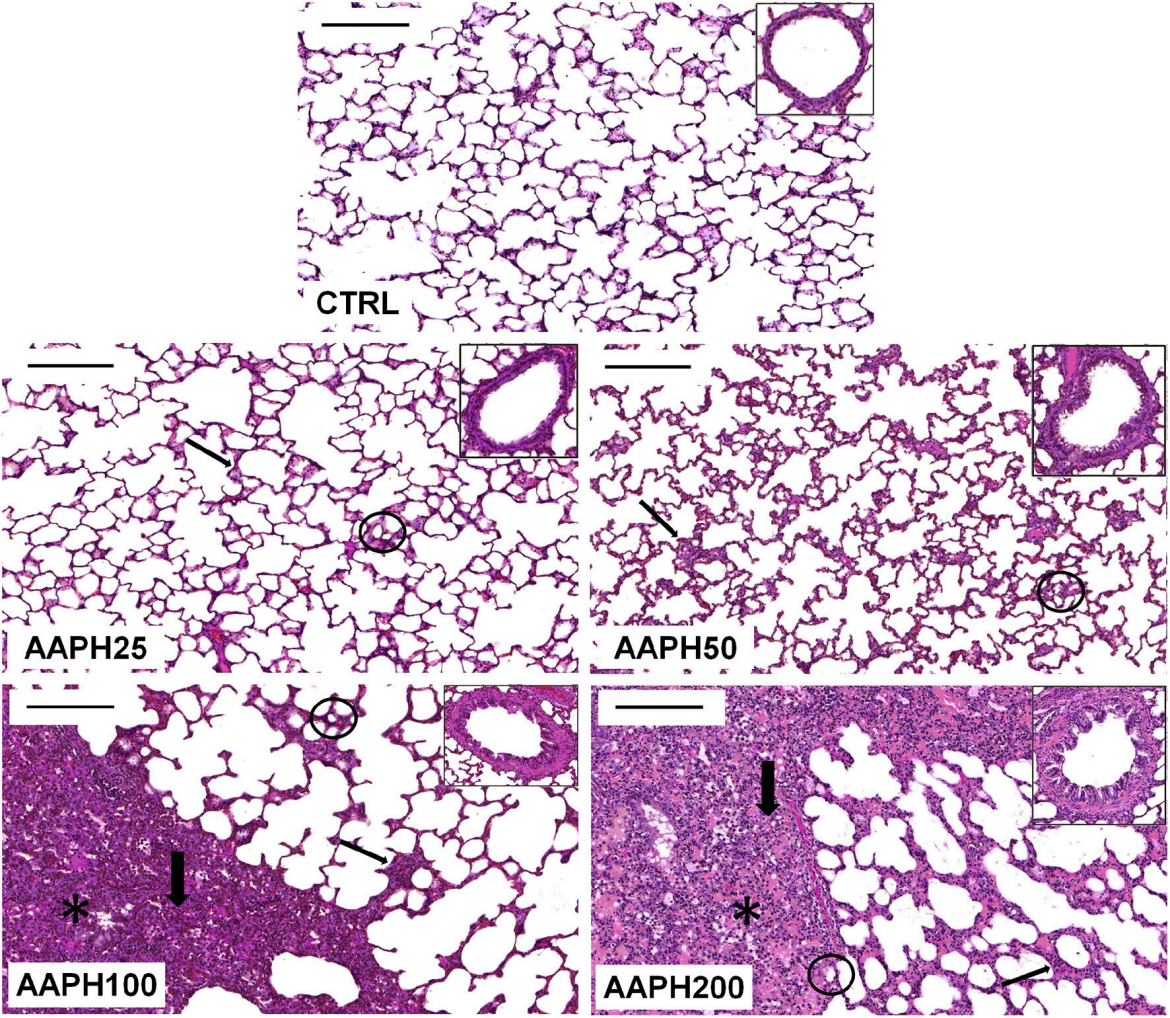
**Photomicrographs of lung parenchyma stained with hematoxylin–eosin**. Measurements were done 72 h after the exposure of animals to saline solution (CTRL) or different amounts of 2,2′-azobis (2-amidinopropane) dihydrochloride (AAPH25, 50, 100, and 200 mg/kg in saline solution). Thin arrows: thickened septa; thick arrows: cellular infiltrate; circles: alveolar collapse; and *: hepatized area. Inserts show representative airways in each group. Bars = 200 μm.

Figure 2 depicts photomicrographs of lung parenchyma showing alveolar collapse, thickened septa, cellular infiltration, and hepatized areas after instillation of AAPH. Additionally, each panel insert shows thickening of the airway epithelium in AAPH animals. However, AAPH100 and AAPH200 groups displayed a more important lung injury than the other AAPH groups. These findings support the presence of an inflammatory process, which probably is correlated with the increase in resistive and elastic lung mechanical parameters (ΔP1 and Est, Figures 1A,D).

**Figure 3:**
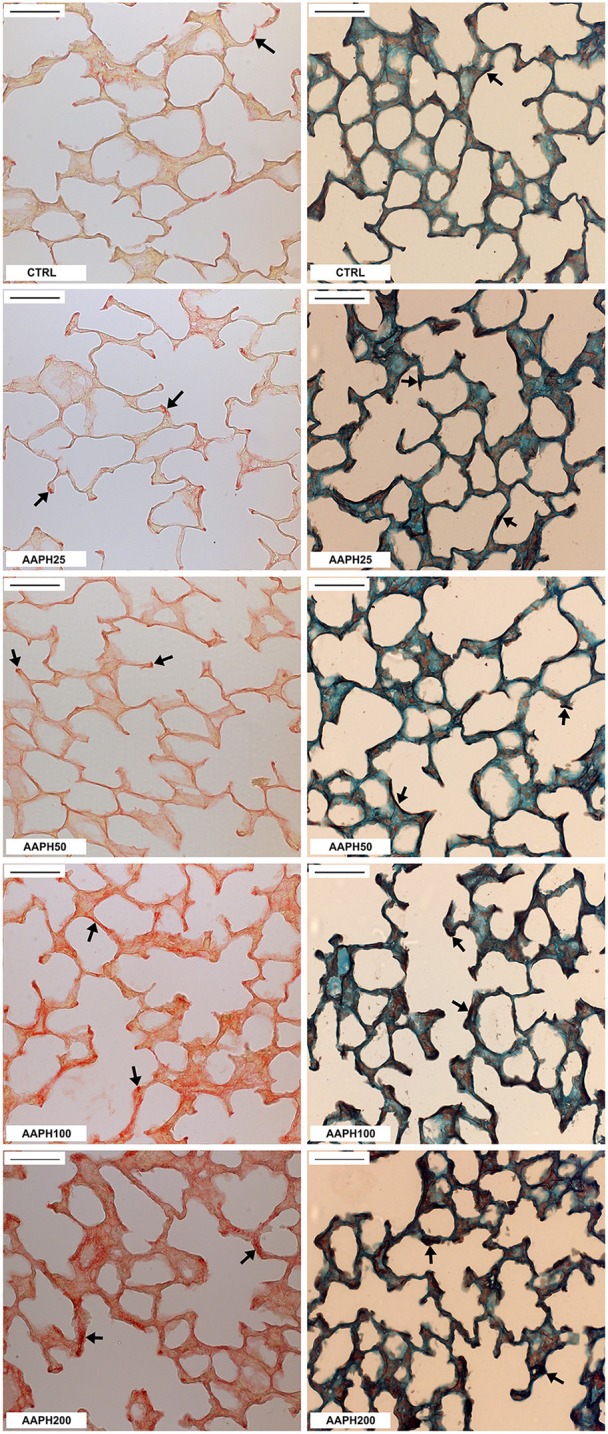
**Collagen and elastic fibers in the lung parenchyma**. Photomicrographs of lung parenchyma showing collagen (left column) and elastic fibers (right column). Measurements were done 72 h after the exposure of animals to saline solution (CTRL) or different amounts of 2,2′-azobis (2-amidinopropane) dihydrochloride (AAPH25, 50, 100, and 200 mg/kg in saline solution). Arrows indicate collagen and elastic fibers. Bars = 50 μm.

The area occupied by collagen fibers was significantly higher in AAPH100 and AAPH200 groups, whereas the area of elastic fibers was significantly higher than CTRL only in AAPH200 group (Table 1; Figure 3). Therefore, alterations in extracellular matrix composition, as expressed by collagen and elastic fiber contents indicate pulmonary remodeling, which is in line with Figure 2.

The number of PMN and MN cells were significantly higher in AAPH200 rats than in CTRL group (Table 1). Regarding PMN influx, it should be pointed out that the significance level between CTRL and AAPH100 was 0.07, and, thus, the results should be cautiously interpreted.

**Figure 4:**
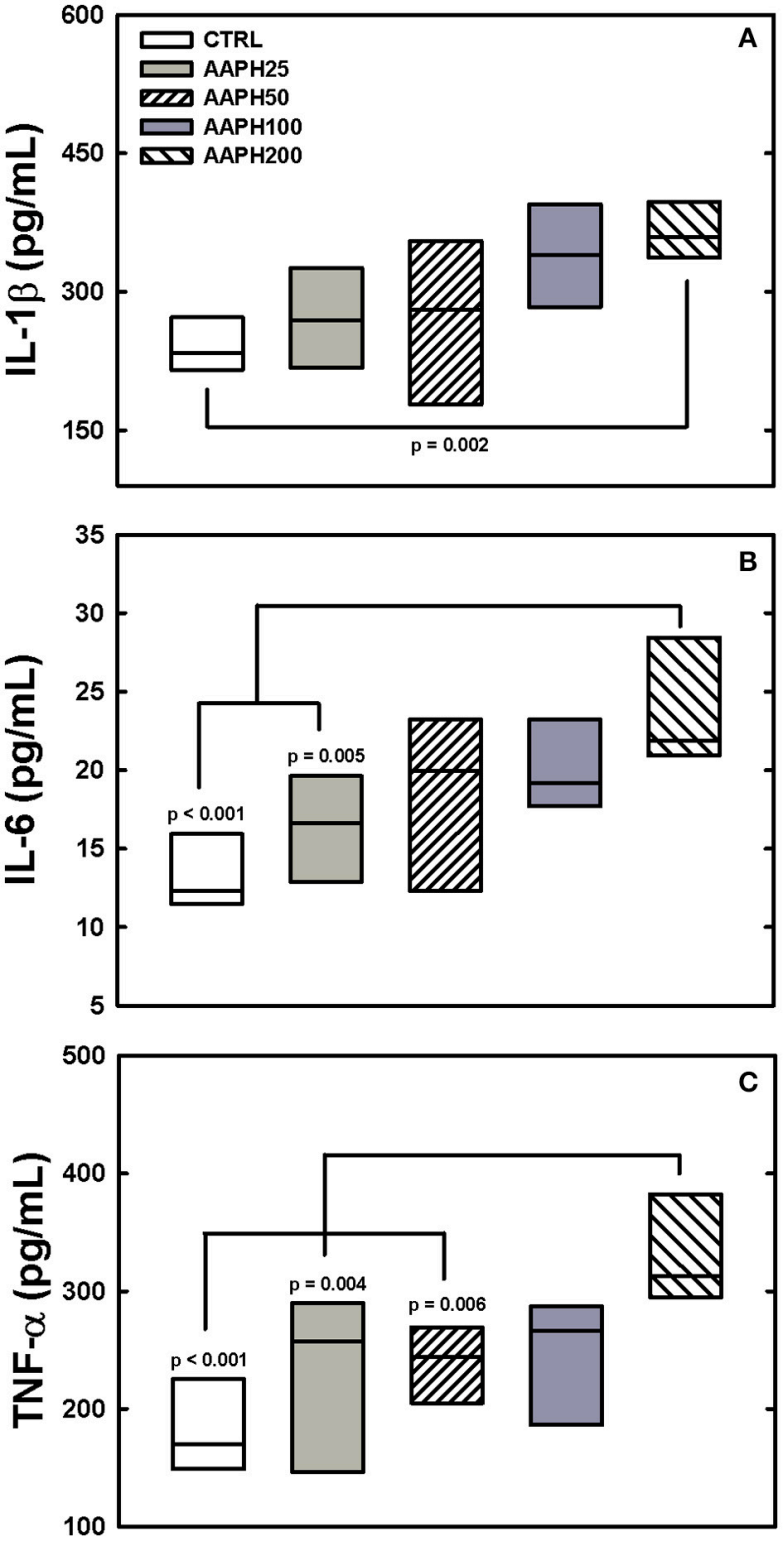
**Levels of citokines**. IL-1β, IL-6, and TNF-α (panels **A–C**, respectively) were measured in lung homogenate by ELISA 72 h after exposure of the animals to saline solution (CTRL) or different amounts of 2,2′-azobis (2-amidinopropane) dihydrochloride (AAPH25, 50, 100, and 200 mg/kg in saline solution). In panel **(A)** AAPH200 IL-1β was larger than in CTRL animals. In panel **(B)** AAPH200 IL-6 was greater than in CTRL and AAPH25 rats that did not differ. Panel **(C)** shows that AAPH200 TNF-α was greater than in CTRL, AAAPH25 and AAPH50, which did not differ among them. Boxes show interquartile (25–75%) range and horizontal lines represent median of 6 animals in each group. *P*-values are shown.

AAPH induced inflammatory changes, as expressed by increased release of pro-inflammatory cytokines IL-1β, IL-6 e TNF-α. IL-1β was significantly higher in AAPH200 group than in CTRL animals (Figure 4A). IL-6 presented larger values in AAPH200 rats than in CTRL and AAPH25 groups (Figure 4B). AAPH200 group also presented higher TNF-α levels than CTRL, AAPH25 and AAPH50 rats (Figure 4C). In all instances, a trend to progressively higher values can be seen between CTRL and AAPH200 rats (Figure 4).

**Figure 5:**
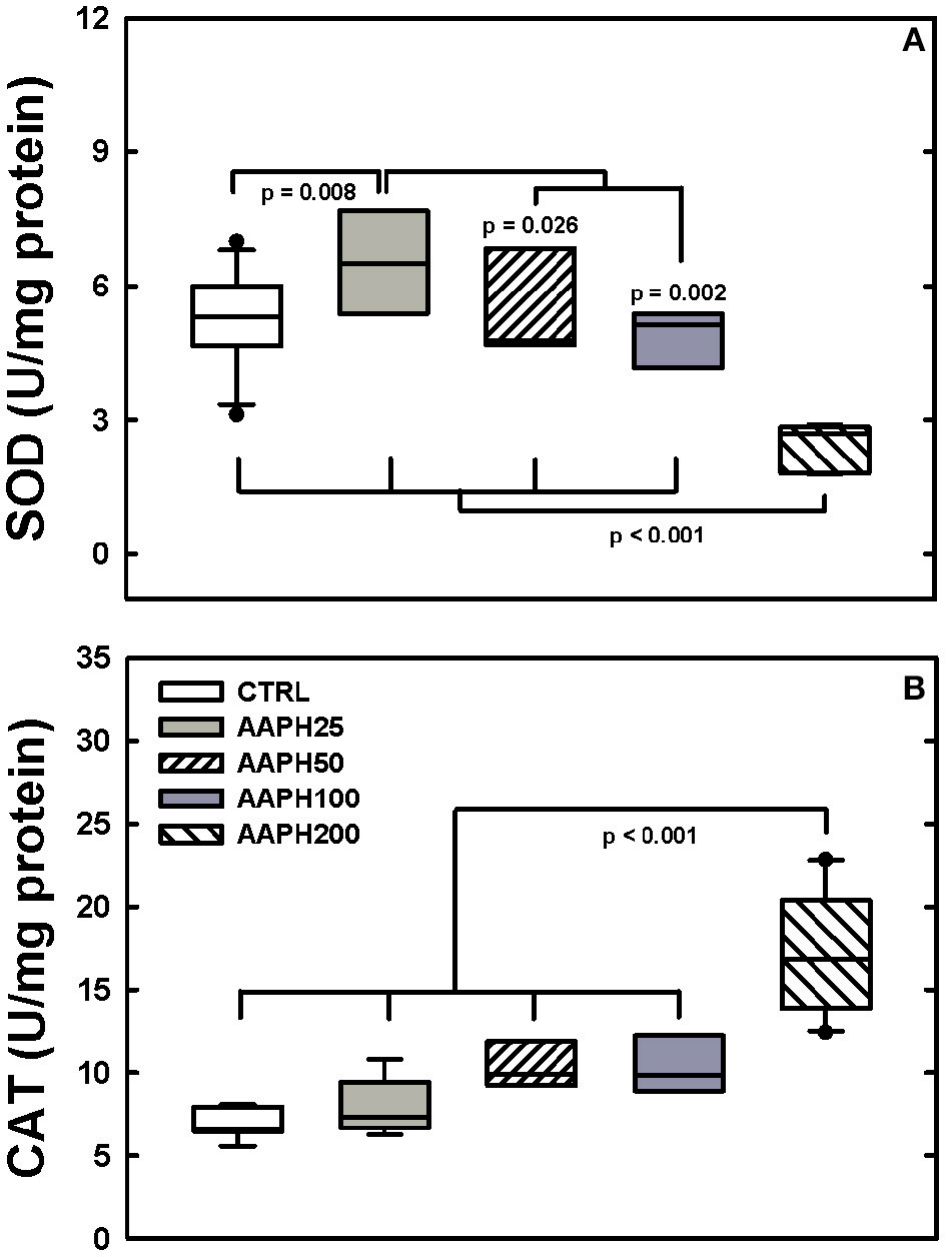
**Determination of enzymatic activity. (A)** Superoxide dismutase (SOD). **(B)** Catalase (CAT). Animals exposed to saline solution (CTRL) or different amounts of 2,2′-azobis (2-amidinopropane) dihydrochloride (AAPH25, 50, 100, and 200 mg/kg in saline solution). Panel **(A)** shows that SOD was smaller in AAPH200 group than in the other rats; AAPH25 presented SOD values larger than in the remaining groups. In panel **(B)** AAPH200 was larger than in the other groups that did not differ among themselves. Boxes show interquartile (25–75%) range, whiskers encompass 10–90% range, and horizontal lines represent median of 8 animals in each group. *P*-values are depicted.

SOD activity was higher in AAPH25 rats than in the remaining groups and AAPH200 rats presented lower values than the other groups (Figure 5A). CAT activity was significantly greater in AAPH200 group than in the remaining groups (Figure 5B).

**Figure 6:**
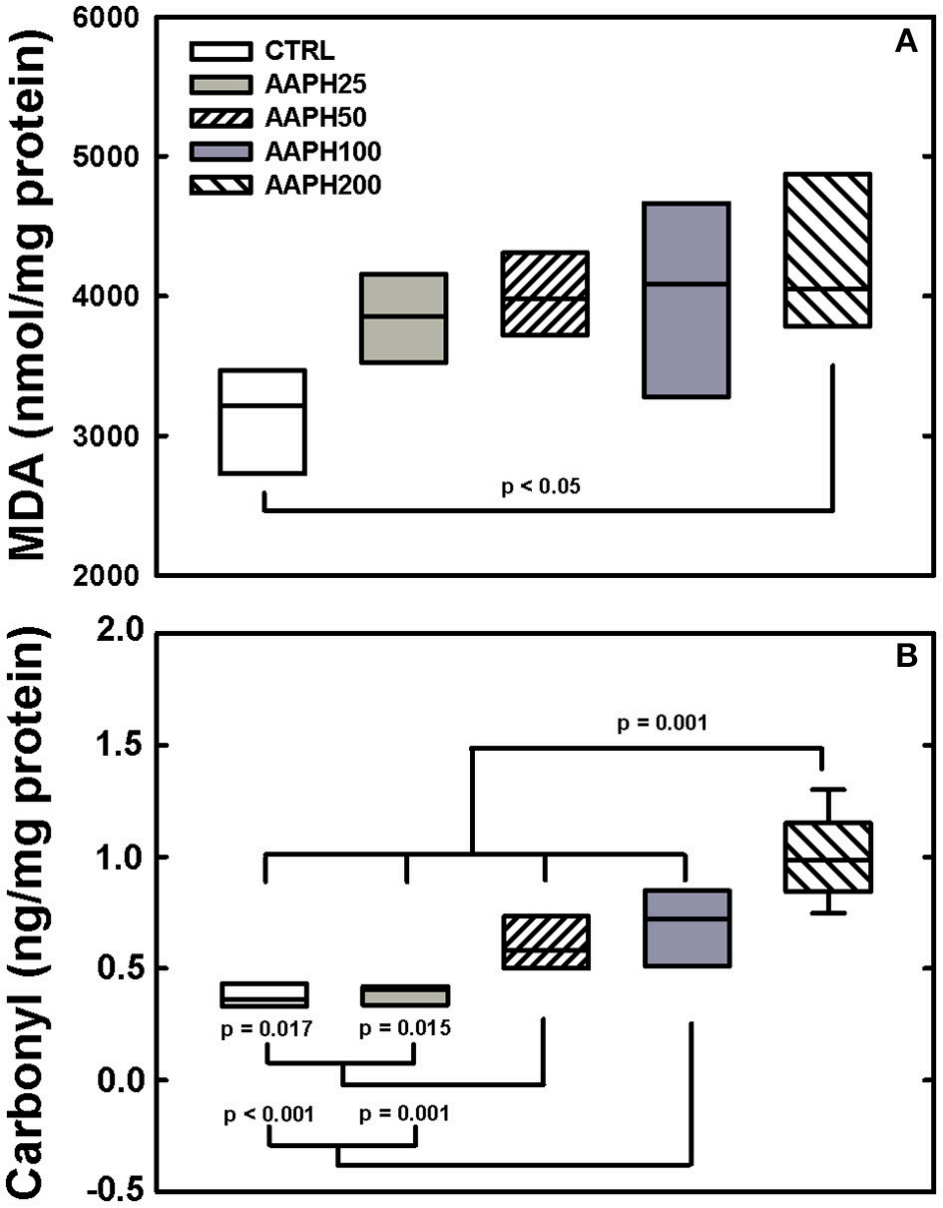
**Determination of oxidative damage. (A)** Malondialdehyde (MDA). **(B)** Carbonyl groups. Animals exposed to saline solution (CTRL) or different amounts of 2,2′-azobis (2-amidinopropane) dihydrochloride (AAPH25, 50, 100, and 200 mg/kg in saline solution). Panel **(A)** shows that AAPH200 presented higher values of MDA than CTRL animals. In panel **(B)**, AAPH200 carbonyl value was larger than in the other groups; AAPH50 and AAPH100 were greater than in CTRL and AAPH25 groups. Boxes show interquartile (25–75%) range, whiskers encompass 10–90% range, and horizontal lines represent median of 8 animals in each group. *P*-values are shown.

Levels of carbonyl groups were largern in AAPH200 rats than in the other groups, AAPH50 and AAPH100 were higher than CTRL and AAPH rats (Figure 6B). Levels of MDA were larger in AAPH200 group than in the remaining groups (Figure 6A).

## Discussion

The aim of the present study was to investigate whether AAPH is able to generate functional, histological, inflammatory, and biochemical impairments in the lung. For such purpose, lung mechanics and histology, as well as redox and inflammatory state were used as markers. Our findings demonstrated that AAPH exposure caused significant alterations *in vivo* in mechanics, together with histologic and inflammatory alterations and redox imbalance in rat lungs.

The literature shows that reactive oxygen species (ROS) can be harmful or beneficial in biological systems depending on the environment (Phaniendra et al., 2015). At high concentrations, ROS can mediate damage to cell structures, including lipids, proteins, and nucleic acids (Rahman, 2007; Valko et al., 2007). Remarkably, it is known that oxidative stress plays a central role in the pathogenesis of various lung disorders such as asthma, lung cancer, pulmonary fibrosis, COPD and ALI in human beings (Valko et al., 2007; Park et al., 2009). Hence, the redox environment is of paramount importance in triggering respiratory disease.

Oxidative stress can be addressed by a variety of agents, including hydrogen peroxide, xanthine oxidase and organic hydroperoxides (Miki et al., 1987; Musialik et al., 2008), but AAPH offers some specific advantages. AAPH generates free radicals by its unimolecular thermal decomposition, which quickly react with oxygen, producing peroxyl radicals (Dooley et al., 1990; Landi et al., 1995; Hanlon and Seybert, 1997; Yokozawa et al., 2000; Zhu et al., 2005). In their turn, peroxyl radicals, which are high-energy species and display biological diversity in their actions, induce lipid peroxidation. The determination of lipid peroxidation is the most frequently used approach to reveal involvement of peroxyl radicals in human disease (Gutteridge, 1995; Cadenas and Sies, 1998). As there is no report in the literature on the use of AAPH intranasally, we conducted pilot experiments and chose the timeline and doses used in this study accordingly.

Respiratory mechanical parameters were measured by the end-inflation occlusion method that allows the identification of elastic, resistive, and viscoelastic and/or inhomogeneous lung mechanical components (Bates et al., 1985, 1988). Our results showed that Est was significantly higher in AAPH100 and AAPH200 than in CTRL rats, indicating stiffer lungs and greater inspiratory impedance (Figure 1). Possibly, the increased elastance is partly secondary to the presence of inflammation of the lung parenchyma, characterized by edema, influx of inflammatory cells, hepatized areas and septal thickening (Figure 2). Indeed, in this context Table 1 shows that there is a significant increase in the amount of polymorpho- and mononuclear cells in AAPH200 group. There is also a trend in the same direction in AAPH100 rats.

In line with our results, evidence suggest that inflammatory processes can potentially impair the synthesis and/or storage of pulmonary surfactant, elevating alveolar surface tension and thus producing alveolar collapse with consequent increase in Est (Jain et al., 1992; Liu et al., 1996). In this context, type II alveolar epithelial cells produce and secrete surfactant proteins to maintain morphological organization, biophysical, and immune functions, and biochemical composition in lung surface film (Lu et al., 2010). The methionine residues in these proteins can be oxidized and inactived by ROS, which diminishes the ability of the surfactant to lower lung surface tension (Chao et al., 1997; Manzanares et al., 2007). Decomposition of AAPH can oxidize amino acid residues in proteins, including methionine, tyrosine, and tryptophan (Chao et al., 1997; Ji et al., 2009). Pro-inflammatory cytokines, such as TNF-α, IL-1β, and IL-6, are involved in both asthma and COPD and may play a role in amplifying inflammation and thus determining disease severity (Barnes, 2008). Indeed, we observed influx of inflammatory cells, alveolar collapse (Figure 2) and increase in pro-inflammatory citokines IL-1β, TNF-α, and IL-6 (Figure 4) in treated rats. Thus, it would be reasonable to hypothesize that lung exposure to AAPH increased Est owing to inflammation and alveolar collapse resulting from the impairment in the synthesis and/or storage of pulmonary surfactant.

Our results also disclossed that higher doses of AAPH significantly increased lung resistive pressure (ΔP1), suggesting that the airways were also affected. This is evidenced by progressive thickening of the airway wall with increasing dose of AAPH (Figure 2), and the bronchoconstriction index disclosed that all AAPH groups similarly displayed reduced lumina (Table 1). Our data corroborate previous studies in organ baths, which evaluate the contractile effect of H_2_O_2_ on rat (Szarek and Schmidt, 1990) and cat (Bauer et al., 1997) airway smooth muscle. Moreover, respiratory diseases are related to increased free radical production in airway smooth muscle, which alters the excitation-contraction coupling, increasing baseline smooth muscle tonus (Hulsmann et al., 1994; Sadeghi-Hashjin et al., 1996; Cortijo et al., 1999; Wiegman et al., 2015).

The amount of elastic and collagen fibers was increased in groups treated with high doses of AAPH (Table 1; Figure 3). A complex mechanism modulate the transcription of molecules that destroy the lung extracellular matrix proteins and produces activation/inhibition of diverse cells of the lung tissue, which altogether regulate matrix remodeling (Rocco et al., 2001). These modifications may alter lung geometry, and, hence, its elastic component (James and Wenzel, 2007). Rocco et al. (2001) demonstrated increased collagen and elastic components in a model of ALI, yielding a process of fibroelastosis that started early in the evolution of the lesion. Therefore, our data are consistent with the literature, since ROS may contribute to the development of pulmonary fibrosis owing to their effects on the production of cytokines and growth factors, such as TGF-β (Park et al., 2009). Additionally, the increase in ROS can stimulate fibroblast migration to injured areas, where they will secrete collagen and other matrix proteins, contributing to the stiffening of lung parenchyma (Kirkham and Barnes, 2013). In fact, oxidative stress plays an important role in the remodeling process, interfering with the architecture and, consequently, with lung compliance, which is observed in patients with asthma, COPD, ALI, and pulmonary fibrosis (MacNee, 2001; Kluchová et al., 2007). Therefore, our finding of increased pro-inflammatory cytokines levels (Figure 4) may explain the augmented deposition of collagen and elastic fibers (Table 1; Figure 3), as well as the increased Est (Figure 1).

In order to assess the redox activity, we analyzed the levels of the antioxidant enzymes SOD and CAT in lung homogenate. SOD activity was significantly augmented in AAPH25 (lowest dose) and diminished in AAPH200 (highest dose). Conversely, CAT activity was significantly increased only in AAPH200 (Figure 5). Indeed, a low level of oxidative stress can stimulate an increase in antioxidant enzyme activity, while a high level can induce protein damage and decreased antioxidant enzyme activity. In this case, a reduced enzyme activity may occur via direct oxidative damage of the enzyme molecules, or via oxidative stress-altered enzymes gene expression, or both (Dreher and Junod, 1996; Comhair et al., 2005; Park et al., 2009). In line with our CAT and SOD activities results in AAPH200 group, Escobar et al. (1996) reported that SOD was readily inactivated by AAPH in *in vitro* assay. Kwon et al. (2000) showed that exposure of SOD to AAPH led to the protein fragmentation, associated with enzymatic inactivation and generation of protein carbonyl (Kwon et al., 2000), which is in line with our protein damage data (Figure 6B). Increased CAT activity in AAPH200 group was probably caused in part due to increased cellular infiltration (Figure 2; Table 1). Consistent with this argument, the main cellular sources of ROS in the lung include neutrophils, eosinophils and alveolar macrophages (Ciencewicki et al., 2008). Increasing cellular infiltration releases ROS, which leads to augmented hydrogen peroxide content, the substrate of CAT. Taken these data together, it is reasonable to infer that the increase in CAT activity may be a compensatory mechanism for the overproduction of free radicals.

In order to assess the oxidative damage induced by AAPH, we measured lipid peroxidation by means of the end product MDA, and protein oxidation by the concentration of carbonyl groups (Figure 6; Valko et al., 2007; Kirkham and Barnes, 2013). Our data show that exposure to AAPH significantly increased lipid peroxidation only in AAPH200. On the other hand, levels of carbonyl groups were significantly higher in AAPH50, AAPH100, and AAPH200. Moreover, MDA can inactivate SOD by oxidizing the histidine residues located in the active site of the enzyme (Koh et al., 1999). Thus, these findings suggest that AAPH-derived free radicals cause oxidative damage by disturbing the antioxidant defense systems (Figure 6). Interestingly, increased levels of carbonyls and MDA were also detected in the lungs of patients with COPD (Kluchová et al., 2007). Additionally, COPD show elevated levels of ROS and carbonyls that may be associated with increased inflammation and airway remodeling (Kirkham and Barnes, 2013).

Our study presents limitations: (1) we did not measure cellularity in the BALF; (2) the induction of prostaglandins involved in the permeability of blood vessel was not determined.

In conclusion, exposure to AAPH caused significant inflammatory alterations and redox imbalance accompanied by altered lung mechanics and histology. Furthermore, we disclosed that exposure to AAPH, especially using the dose of 200 mg/kg, may represent a useful *in vivo* tool to trigger lung lesions.

## Author contributions

Substantial contributions to the conception or design of the work: MM, GC, JL, SV, and WZ. Substantial contributions to the acquisition, analysis, or interpretation of data for the work: MM, GC, SV, AA, NC, and WZ. Drafting the work or revising it critically for important intellectual content: MM, GC, JL, SV, NC, and WZ. Final approval of the version to be published: MM, GC, JL, SV, NC, AA, and WZ. Agreement to be accountable for all aspects of the work in ensuring that questions related to the accuracy or integrity of any part of the work are appropriately investigated and resolved: MM, GC, JL, SV, NC, AA, and WZ.

## Funding

This work was supported by the “Fundação Cearense de Apoio ao desenvolvimento Científico e Tecnológico-FUNCAP” (grant 10430447-2); Conselho Nacional de Desenvolvimento Científico e Tecnológico e Inovação-CNPq (grants 300531/2012-5, 470495/2012-0); Fundação Carlos Chagas Filho de Apoio à Pesquisa do Estado do Rio de Janeiro-FAPERJ (grants E-26/112.092/2012, E-26/201.450/2014), Brazil. The funders had no role in study design, data collection and analysis, decision to publish or preparation of the manuscript.

### Conflict of interest statement

The authors declare that the research was conducted in the absence of any commercial or financial relationships that could be construed as a potential conflict of interest.
